# Approach to the Performance of Polymers Designed Based on Poly(methyl methacrylate) (PMMA)/poly(urethane) (PU) with Recycled Cellulose Nanoparticles from Cold Drink Cups

**DOI:** 10.3390/polym17091141

**Published:** 2025-04-22

**Authors:** Erick Habacuc Reyes Piña, Mayra Elizabeth Juárez Méndez, Diana Palma Ramírez, Acela López Benítez, Karen Ailed Neri Espinoza, Nicolás Cayetano Castro

**Affiliations:** 1 Department of Polymers and Nanomaterials, Unidad Profesional Interdisciplinaria de Ingeniería Campus Hidalgo (UPIIH), Instituto Politécnico Nacional (IPN), San Agustín Tlaxiaca 42162, Hidalgo, Mexico; ereyesp1901@alumno.ipn.mx (E.H.R.P.); aclopezb@ipn.mx (A.L.B.); kneri@ipn.mx (K.A.N.E.); 2 Department of Chemical and Biochemical Engineering, Tecnológico Nacional de México (TecNM), Ciudad Madero 89460, Tamaulipas, Mexico; mayra.jm@cdmadero.tecnm.mx; 3 CNMN, Instituto Politécnico Nacional, Wilfrido Massieu s/n, UPALM, Gustavo A. Madero, Mexico City 07738, Mexico; ncayetanoc@ipn.mx

**Keywords:** interpenetrating polymer networks, headlights, PMMA, PU, crystalline cellulose, aging exposure, automotive

## Abstract

Transparent high-performance polymers are essential to avoid damage in automotive headlights when exposed to environmental conditions. An approach involving the synthesis of reinforced interpenetrating polymer networks (IPNs) based on poly(methyl methacrylate) (PMMA)/poly(urethane) (PU) with crystalline cellulose (C) is here proposed. The valorization of single-use cups used for cold beverage applications into reinforcement nanoparticle agents is studied through structural and morphological analysis, revealing intermediate crystallinity (52.51%) with a mixture of Iα (52.9%) and Iβ (46.3%) polymorphs in which the initial fiber had no chemical modification after the involved pretreatments. The effect of dispersing 0.1 wt% of C (d = 29 nm and L= 85–200 nm) into 50/50 and 80/20 PMMA/PU ratios is studied as a reinforcement agent under aging and environmental conditions (ASTM D1435-20) for 672 h. PMMA_80_/PU_20_ (σ =4 MPa, ε = 54%, E = 7 MPa) led to lower mechanical properties than PMMA_50_/PU_50_ (stress σ =14 MPa, strain ε = 94%, E = 83 MPa). PMMA_50_/PU_50_/C is reinforced in σ and ε with C addition (σ = 19 MPa, ε = 41%, E = 585 MPa) while PMMA_80_/PU_20_/C reduces both (σ = 3 MPa, ε = 51%, E = 5 MPa). This study indicates that aging increases stress while maintaining strain in the first but decreases in the second. The optical properties indicate no severe damage after aging.

## 1. Introduction

The automotive industry is currently interested in developing new quality engineering materials that consider low production costs and fuel economy [1]. To date, different types of materials have been used, including ceramics, metals, and polymers. However, their use depends on the application and the area within the vehicle where they are located [2].

An important factor in most road accidents is the condition of the vehicle and the user’s driving skills. Within the first category, the condition of its components is relevant, for example, in the case of automotive headlights, as they can present some drawbacks regarding durability due to environmental damage. Headlights were once fabricated with glass, and polymer-based materials are now used for their fabrication [3].

Polymer materials have revealed several issues, including chemical attacks on the inner and outer surfaces of the automotive part, which cause fogginess, haziness, cloudiness, or white patches. This can be due to oxidation, flying debris, dirt, water vapor, and chemical attacks, to mention a few [3]. Among the most commonly used polymers for lightweight, thermally stable, and scratch-resistant applications in the manufacture of headlights, polymethyl methacrylate (PMMA) and polycarbonate (PC) polymers are favored due to their optimal optical and mechanical properties. PMMA stands out for its excellent transparency, UV resistance, and long-term stability, making it ideal for applications where optical clarity is required [4]. However, its impact resistance is limited and deteriorates when exposed to chemicals. On the other hand, a PC is a material that is more resistant to impact and thermal. However, it suffers more significant degradation when exposed to ultraviolet (UV) radiation, which can compromise its transparency over time [5]. For these reasons, exploring options that minimize wear and tear during use is essential.

An alternative approach has been to incorporate nanoparticles, such as nano-SiO_2_, directly into the polymer formulation. However, this can lead to the final product becoming opaque, which reduces its functionality. Therefore, new methods need to be explored. A promising solution to overcome these limitations is to apply coatings such as silicone to the surface of headlights [6]. Following this trend, this work proposes the use of high-performance interpenetrating polymer networks (IPNs) as coatings for automotive headlight applications. IPNs are a class of polymer materials that offer enhanced functionalities compared to individual network polymers [7]. A practical approach to achieving a transparent polymer with superior mechanical properties involves blending polyurethane (PU) with PMMA to develop engineering polymers suitable for processing into IPNs. PU is a versatile polymer renowned for its flexibility, high impact resistance, durability, and damping properties [8]. It also provides excellent wear resistance and stability under adverse weather conditions [9].

Incorporating reinforcing particles, such as crystalline cellulose, into the interpenetrating networks of PU and PMMA can enhance both the mechanical and optical properties [10]. Cellulose, which is extracted from renewable sources, is notable for its high rigidity, mechanical strength, and transparency, making it suitable for optical applications such as automotive headlights [11]. When added to the polymeric matrix, cellulose creates a nanometric-reinforced structure that increases the material’s impact resistance, hardness, and dimensional stability without sacrificing optical clarity [12]. This additional reinforcement not only enhances the material’s durability but also enhances its performance under extreme environmental conditions, positioning it as an efficient and sustainable alternative for use in advanced automotive headlights [13].

This study focuses on producing polymers in an IPN form based on crystalline cellulose particles derived from recycled single-use cups, along with PMMA and PU polymers. The functional groups of the PMMA/PU/cellulose polymers are examined to confirm polymerization and evaluate their emission properties, which helps in assessing the dispersion of cellulose. Under real environmental conditions, as per a previous study, the aging process is analyzed through structural, mechanical, and optical analyses. This research aims to propose these polymers as a method to extend the useful life of automotive headlights and to explore their potential application as protective coatings.

## 2. Materials and Methods

Single-use cups from the ICEE company were used as raw materials for cold beverages. To separate the coating from the fiber, a handmade hydrapulper operated at 2000 rpm was employed, using 1 L of deionized water (Wohler, Pottstown, PA, USA) and 500 g of the cups. The fiber was filtered from the coating and subsequently dried in an oven at 60 °C for 24 h. Then, the extractives were removed from the fiber by soaking 40 g with 500 mL of absolute ethanol (99.9%, J.T. Baker, Phillipsburg, NJ, USA) for 4 h. The fiber was then pretreated with a solution consisting of 400 mL of 10% (*w*/*v*) of sodium hydroxide (≥98.0%, J. T. Baker) and 10% (*v*/*v*) of hydrogen peroxide (30%, Wohler, Middleton, MA, USA) while being stirred magnetically at room temperature. Following this, the fiber was filtered and neutralized. This pretreatment process was repeated, and the fiber was dried again at 50 °C for 24 h. Acid hydrolysis was performed using 64% (*v*/*v*) sulfuric acid (98–98%, Fermont, Monterrey, Mexico), considering 1 g fiber/8.75 mL of solution at 45 °C for 30 min under magnetic stirring. Cold deionized water (1 g fiber/16 mL) was added to stop the reaction. The sample was then placed in a dialysis bag to remove low-molecular-weight compounds. These compounds were diluted in deionized water, which was changed daily until neutralization was achieved. Finally, the samples were dried at 50 °C for 24 h.

A mixture of castor oil (0.0012 eq., Sigma-Aldrich, St. Louis and Burlington, MA, USA, 100%) with 0.012 eq. 1,1,1 (trihydroxy) methylpropane (Sigma-Aldrich, 90%) was homogenized at 80 °C for 3 h before creating the three-dimensional networks of interpenetrated networks. Subsequently, the following raw materials were combined at room temperature: methyl methacrylate (MMA) monomer (Sigma-Aldrich, 99%), hexamethylene diisocyanate monomer (Sigma Aldrich, ≥98%), trimethylolpropane trimethacrylate (Sigma Aldrich, 90%), and benzoyl peroxide (Sigma Aldrich, 99%), as an initiator. The mixture was stirred for 3 h at room temperature while preventing evaporation. Dibutyltin dilaurate (Sigma-Aldrich, 95%) was added as a catalyst at the end of this process. The resulting solution was poured into a glass mold, allowing it to polymerize for 24 h in a vacuum desiccator to prevent moisture absorption. Subsequently, the mixture was exposed to UV light of a wavelength of 365 nm for 3 h before being demolded. Ratios of 50/50 and 80/20 of PMMA/PU were considered for the formulation. The crystalline cellulose particles were added during the 3 h polymerization at 0.1 wt% followed by ultrasonic treatment to generate an homogeneous dispersion. This ultrasonic processing was conducted in cycles at 42 kHz for 1 h, and magnetic stirring was performed for an additional hour. The catalyst was then added at the end of the cycle. The mixture was kept under vacuum to remove air and moisture before pouring into the mold. Finally, the mold was placed under UV light (365 nm) for 3 h.

A Bruker ALPHA II spectrometer (Bruker, Billerica, MA, USA) was used to analyze the functional groups present in the samples. This spectrometer has a spectral range of 4000–650 cm^−1^ and a resolution of 2 cm^−1^, featuring a diamond crystal for the attenuated total reflectance (ATR) accessory. To investigate the crystallinity and structural presence of polymorphs in the crystalline cellulose sample, a D2 phaser, from Bruker, was employed. This device operated within the range from 10 to 40° (2θ) using CuK_α_= 0.154 nm radiation at 40 kV.

To evaluate the morphological features, a VELAB^®^ VE-BC1 optical microscope equipped with an achromatic optical system, multiple objective lenses (4×, 10×, 40×, and 100×), and an integrated 3MP camera was used. The instrument features a dual-plate stage with coaxial movement and a range of 135 mm. The samples were deposited onto microscope slides. Once prepared, the slides were placed onto the microscope stage. The eyepieces were adjusted to their initial position, and the appropriate objective lens was selected to initiate the observation. During the initial visualization, both individual fibers and fiber agglomerates were identified. These preliminary observations were documented through image capture and subsequently processed using specialized software, imageJ version 1.54p, for evaluating the diameter of particles of the significant length. This procedure was systematically repeated for each of the sample preparation conditions.

A LSM 700 confocal scanning laser microscope (CLSM) from Carl ZEISS (Oberkochen, Germany) was used to confirm the emission of crystalline cellulose, assess its incorporation into the polymer samples, and evaluate dispersion.

UV–vis spectra were recorded using a Perkin-Elmer Lambda 40 spectrometer (PerkinElmer, Shelton, CT, USA), which is equipped with an integrating sphere. The spectralon SRS-99-010 (99% reflectance) was the reference for UV–vis measurements.

Specimens for tensile testing were cut according to the EN-ISO-527-1 standard using Bunmina (Madrid, Spain) brand laser cutting equipment (model LSI6400 003P6). The resulting specimens measured 100 mm in total length, 1 mm in thickness, and 5 mm in gauge length. For compression, diameters of 20 mm and 15 mm in thickness were used for the samples. Tensile and compression tests were performed in an AGS-X universal equipment (Shidmadzu, Kyoto, Japan) at 20 mm min^−1^.

The natural aging process was carried out following the ASTM D1435-20 standard. This standard facilitates controlled and extended exposure of polymers with specified dimensions, allows for the control of the direction of solar radiation, and assesses the stability of the properties of plastic materials. To ensure optimal exposure control and monitoring, a rack that is adjustable to different angles was designed, enabling modification of the position of the plastic materials during the process. The standard specifies that the rack material must be inert and allow air recirculation; therefore, slotted aluminum was chosen for this purpose. The samples were subjected to natural weathering for a total of 672 h.

To monitor temperature, humidity, and UV solar radiation following the ASTM D1435-20 standard, an Arduino Mega 2560 microcontroller was used along with the DTH-11 sensors for temperature and humidity and ML8511 for measuring UV solar radiation. During an exposure period of 672 h, the recorded measurements were as follows: 7–20 ± 2.9 °C, 62.25 ± 6.18%, and 64.82 ± 1.89 mW/cm^2^.

## 3. Results

### 3.1. Cellulose Extraction from Cold Drink Cups

#### 3.1.1. Structural Analysis Through FTIR Spectroscopy

**Figure 1:**
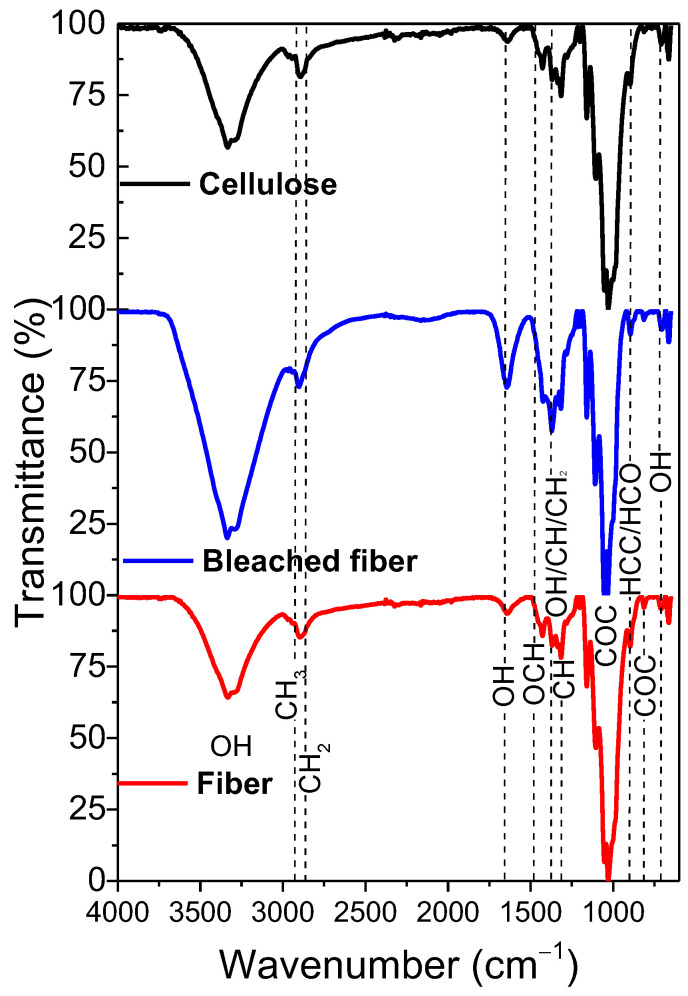
FTIR spectra of the crystalline cellulose extraction.

FTIR is a non-destructive technique that characterizes both covalent and non-covalent interactions in cellulose [14]. It is particularly useful for characterizing water sorption through the hydroxyl and carboxyl groups found in cellulosic polymers [15]. In Figure 1, the FTIR spectrum of cellulose fibers is displayed, along with the spectra of compounds obtained after chemical treatments that yield bleached fiber and crystalline cellulose. The spectrum of the fiber reveals a prominent band at 3335 cm^−1^, which is attributed to the O-H stretching vibration of cellulose and adsorbed water [16]. The stretching vibration of methylene (-CH_2_), confirming that the main component of cups is cellulose, is detected at 2901 cm^−1^ [17]. Water adsorption is further confirmed by a weak band at 1640 cm^−1^, corresponding to the O-H bending vibration [18]. There are also three low-intensity bands at 1430 cm^−1^, 1370 cm^−1^, and 1315 cm^−1^, corresponding to OCH bending in the plane, OH deformation, CH deformation bending in the plane, and CH_2_ deformation, as well as CH deformation rocking, characteristic of cellulose [19]. The contributions between 920 and 1190 cm^−1^ can be associated with the CH/OH/CCH_2_ deformations and CC/COH/COC stretching [19] but mainly with the C-O-C glycosidic bond vibrations [20,21]. Finally, the bands at 897, 814, and 705 cm^−1^ are associated with the bending mode for HCC and HCO at C(6), the asymmetric stretching vibration of C-O-C, and the C-O-H out-of-plane bending, respectively [22,23].

After the bleaching treatment of fiber, the most significant change occurs in the band associated with the O-H groups at 3335 cm^−1^. This band becomes more intense due to the removal of pigments, hemicellulose, and lignin from the fiber. Additionally, this increase is partly attributed to enhanced water adsorption following the bleaching treatment. However, a decrease in the intensity of this band is observed after the extraction of crystalline cellulose. This reduction can be linked to acid hydrolysis, which removes the amorphous regions of the fiber, thereby decreasing the number of O-H groups present [22]. It is crucial to note that after both treatments, the vibrational modes of cellulose remain intact, indicating that these processes allow for effective extraction without altering the functional groups of the cellulose structure.

#### 3.1.2. Polymorph Contribution of Cellulose Through XRD

**Figure 2:**
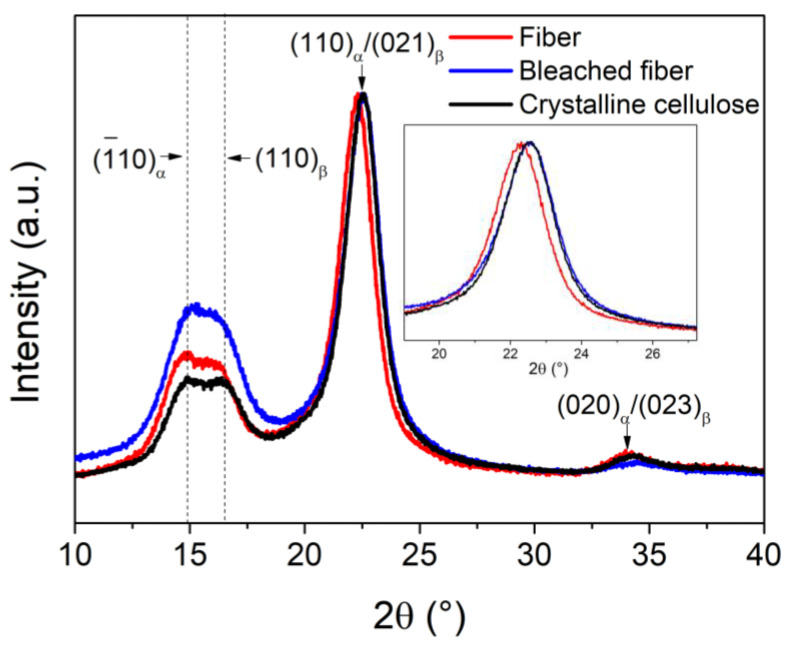
XRD pattern of the crystalline cellulose extraction.

Figure 2 displays the X-ray diffraction of the fiber, the bleached fiber, and crystalline cellulose obtained from single-use cups. The fiber pattern displays the primary signal at 22.3°, which may correspond to both (110) and (021) planes of Iα (triclinic) (PDF # 00-056-1719) and Iβ cellulose (PDF # 00-056-1718), or it could be a result of overlapping signals from both phases. Additionally, there are signals at 14.76° and 16.38°, corresponding to (110) and (110) of Iβ, respectively, along with an overlapping (010) plane from the Iα phase. There is also a broad signal of very low intensity at 33.96°, which corresponds to the overlapping (020)/(023) planes of the Iα and Iβ phases. After the bleaching process, the most notable change is the peak shift to a higher 2θ value, which has been reported in type I cellulose after a more intensive treatment of kenaf fiber [24]. The remaining signals did not show significant changes. Furthermore, the crystalline cellulose also exhibited a slight shift to a higher 2θ value. H. Poshtiri et al. [25] noted that the intensity and sharpness of the signals suggest a high degree of crystallinity, which is directly attributed to the acid hydrolysis process. In particular, the signal detected around 22.3°, corresponding to the (200) diffraction plane, is significantly sharper in the crystalline cellulose sample, indicating a considerable increase in crystallinity. The analysis of the diffraction signals indicates that crystalline cellulose has a crystallinity of 52.51%. It consists of type Iα (triclinic), Iβ (monoclinic), and type II (monoclinic) cellulose in proportions of 52.9%, 46.3%, and 0.8%, respectively. In comparison to the study of L. Wang et al., which reported a decrease in crystallinity due to the transformation from type I to type II type cellulose in fibers based on used disposable cups, the method used in this work shows an increase in crystallinity after pretreatments [26]. Additionally, research on the dissolution and regeneration of cellulose-based films from used disposable paper cups has shown reduced crystallinity [27,28]. It is important to note that these authors focused only on changes in the XRD curves without assessing the actual degree of crystallinity. Z. Kassab et al. found that incorporating cellulose nanocrystals resulted in strong, flexible biomaterials with superior mechanical properties [29]. Therefore, it is likely that crystalline cellulose can enhance the IPNs of PMMA/PU.

#### 3.1.3. Morphological Analysis Through Optical and CLSM Microscopy

Optical microscopy was chosen primarily due to the availability of the equipment, as well as its practicality and relative ease in capturing images of various samples. This technique enables the acquisition of clear and precise images of the fibers, offering sufficient resolution to visualize the morphological characteristics of the analyzed specimens.

**Figure 3:**
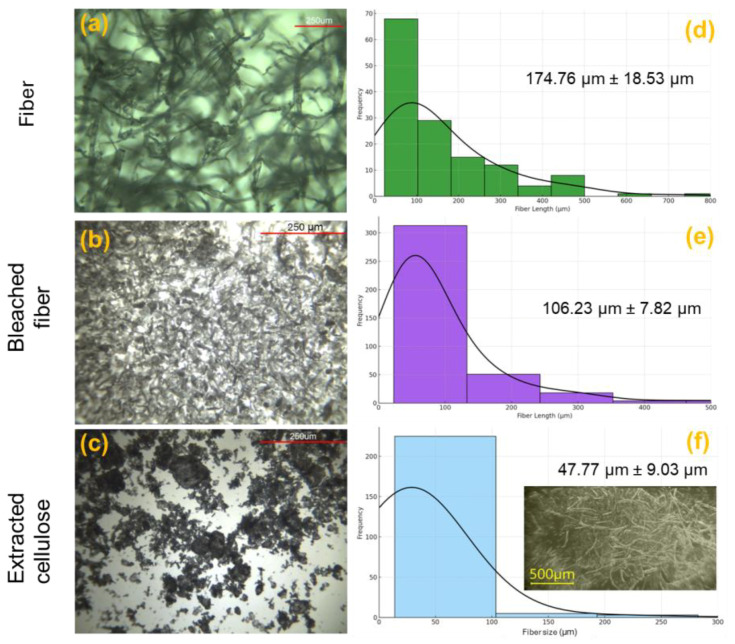
Optical micrographs of (**a**,**b**) fiber, (**c**,**d**) bleached fiber, and (**e**,**f**) extracted cellulose with their respective particle size distribution profiles and average sizes.

Figure 3 shows the evolution of morphology and size distribution of the extracted cellulose materials throughout the disintegration process. Figure 3a,c,e, correspond to optical micrographs at a 250 µm scale, while Figure 3b,d,f represent the corresponding particle or fiber size distributions with fitted curves and average values with uncertainty. Finally, the micro-level morphology of the crystalline cellulose in the micrograph shows a less homogeneous distribution of shorter fibrils, with an average length of 47.77 ± 9.03 µm, reflecting advanced fibrillation and fragmentation. This is due to the hydrolysis process, which apparently reaches a low particle size. The analyzed inset figure also displays an average fiber length of 280 μm with a diameter of 28 μm. This progressive transformation demonstrates the effectiveness of the applied disintegration method and its impact on the morphological characteristics of the cellulose structures. Microcellulose has shown sizes less than 1000 µm [30]. Following the morphological study, it is vital to complement the analysis with CLSM and TEM analyses to provide a detailed understanding of the fiber constitution and relate it to the dispersion within the polymer and the structural properties after aging.

**Figure 4:**
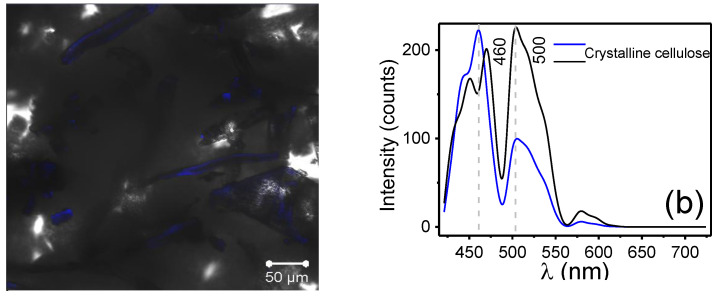
(**a**) CLSM micrograph and (**b**) emission spectrum of crystalline cellulose particles.

Figure 4a,b shows the CLSM micrograph and the emission spectrum of crystalline cellulose. The analysis revealed that the morphology of fibers of varying sizes emits in the visible spectrum, exhibiting with two prominent signals: one consisting of two overlapping peaks between 420 and 490 nm (at 450 and 470 nm, respectively) and the other at 500 nm. These emissions are attributed to the autofluorescence of cellulose fibers, as reported by M. A. Hobisch et al. [31]. Additionally, signals at 522 nm and 574 nm were recorded, which are related to the specific structure of cellulose. These signals arise from the glycosidic bonds between glucose molecules [32]. The fibers have an average length of 115 µm and an average diameter of 20 µm.

#### 3.1.4. Dispersion Analysis of Crystalline Cellulose Through Morphological Analysis

**Figure 5:**
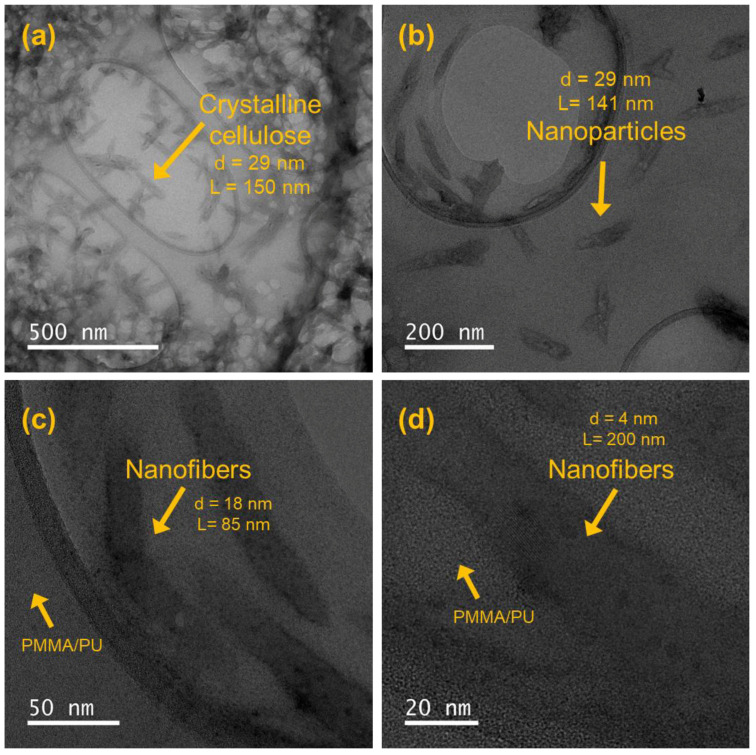
TEM micrographs of crystalline cellulose embedded into PMMA/PU showing nanoparticles with sizes of (**a**) d = 29 nm and L = 150 nm, (**b**) d = 29 nm and L = 141 nm, (**c**) d = 18 nm and L = 85 nm, and (**d**) d = 40 nm and L = 200 nm.

The TEM micrographs presented in Figure 5 reveal a well-dispersed network of cellulose nanofibers embedded within the PMMA/PU sample, demonstrating successful integration and effective processing. The fibers exhibit a high aspect ratio, with consistent nanoscale diameters and lengths extending into the micrometer range. The micrographs indicate minimal fiber agglomeration, suggesting efficient dispersion and positive interaction between the cellulose and polymer matrix. This is likely attributed to favorable interactions between the polymer and fiber, such as hydrogen bonding or mechanical interlocking.

The crystalline cellulose fibers exhibit consistent morphology and high structural integrity, showing no significant signs of fiber breakage or shortening, even at higher magnifications. This uniform and defect-free microstructure indicates efficient stress transfer and potential reinforcement effects within the polymer composite. The polymer matrix tightly encapsulates the fibers, confirming favorable interfacial interactions, possibly through hydrogen bonding between the hydroxyl-rich cellulose surfaces and polar functionalities in the polymer matrix [33].

The TEM micrographs of the PMMA/PU/cellulose samples reveal a cohesive and dense structure, showing no significant voids or microcracks. Although there may be minor fiber agglomeration due to natural hydrogen bonding interactions, these are minimal and are not expected to negatively affect the mechanical performance, which will be analyzed in the next section. Overall, the TEM micrographs indicate that the cellulose nanofibers are effectively dispersed within the matrix, providing optimal reinforcement and enhancing the mechanical properties and overall stability of the polymer composites [34].

In this case, the synthesis of IPNs enhances the mechanical properties by allowing the former polymers to interlock. When a force is applied through the material, stresses are effectively distributed. This means that the load can be shared between both phases, which helps prevent localized stress concentrations that could lead to failure. Typically, the interlocking occurs at the nanometric scale. Therefore, future studies using nanoindentation should be conducted to understand the relationships between particles and entangled networks of polymers, enabling the optimization of architectures for various applications [35,36,37]. Additionally, it is essential to evaluate different ratios of PMMA and PU in future studies to examine how composition affects the nanostructure.

**Figure 6:**
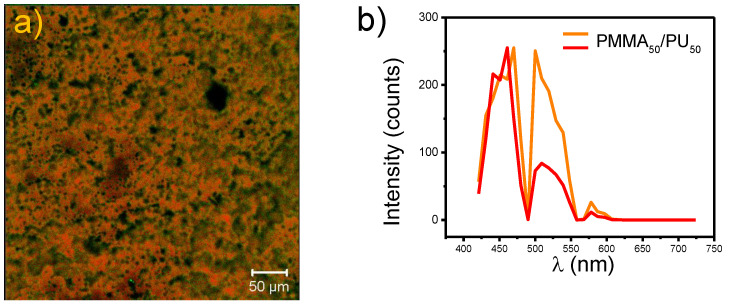

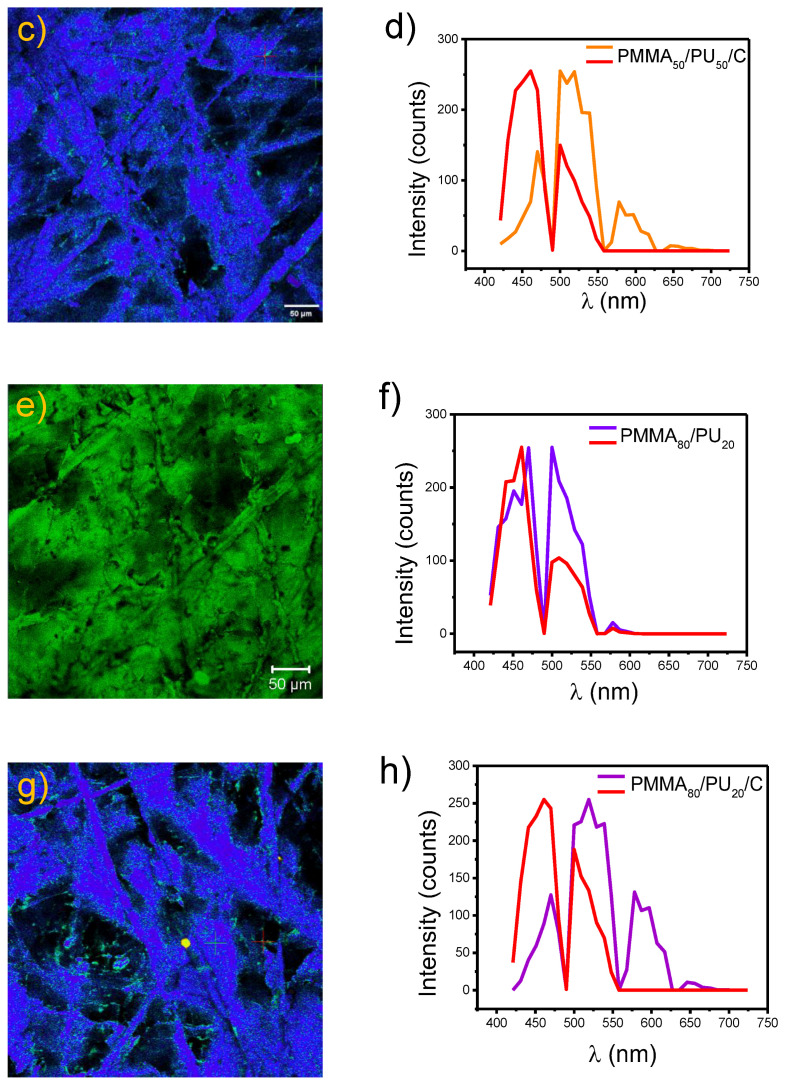
CLSM micrographs of (**a**) PMMA_50_/PU_50_, (**c**) PMMA_50_/PU_50_/C, (**e**) PMMA_80_/PU_20,_ and (**g**) PMMA_80_/PU_20_/C IPNs with their emission spectra in (**b**), (**d**), (**f**) and (**h**), respectively.

Figure 6a shows the CLSM micrograph, where pure PMMA_50_/PU_50_ film surface layers are observed. A rough texture of the material is visualized, forming multi-dimensional circular patterns. The PMMA/PU sample emits in the visible region (Figure 6b). C. Zeng et al. [38] successfully visualized the morphology of different PMMA mixtures. They found that the circular shapes are typical of the polymer, with cells of dimensions approximately 17 µm, similar to the results presented here. When analyzing the incorporation of crystalline cellulose in the polymer to obtain PMMA_50_/PU_50_/C (Figure 6c), particles are found over the entire surface. PMMA_50_/PU_50_/C presents fluorescence at 450, 500, and 575 nm (Figure 6d). When compared with the dimensions and color previously observed in the crystalline cellulose particles, multiple particles exhibiting fluorescence are identified. Thus, these elements correspond to cellulose. It is noteworthy that the dimensions correspond to smaller sizes than those previously recorded. Therefore, it is estimated that the ultrasound process allows for reducing agglomerated fibers and minimizing the final size of the fibers, thereby obtaining small particle sizes.

The pure PMMA_80_/PU_20_ micrograph (Figure 6e) exhibits similar morphological features, resulting from the same polymerization process, in which a physical union is formed within the interpenetrated networks. Clusters of different sizes are visible on the surface, exhibiting a uniform distribution within the generated circular pattern, which shows fluorescence at the indicated location (Figure 6f). C. Zhang. et al. [39] identified the dimensions of the porosity present in PU films, where microporosities covering lengths from 3 to 12 µm stand out, similar to those observed on the surface, with a more significant presence of larger circular shapes, typical of PMMA. This result was expected due to the difference in the ratio of PMMA and PU compared to the PMMA_50_/PU_50_ films, as the latter tends to be more entangled. Dispersing crystalline cellulose to generate the PMMA_80_/PU_20_/C tends to produce some agglomerations of particles (Figure 6g), with the fluorescence contributions of the three phases reaching similar maximums (Figure 6h). Thus, crystalline cellulose was successfully identified in the PMMA/PU samples through fluorescence, which will be useful to relate it to the mechanical performance during aging.

### 3.2. Structural, Optical, and Mechanical Assessment of PMMA/PU/Cellulose IPNs

#### 3.2.1. Structural Analysis of PMMA/PU/Cellulose IPNs

**Figure 7:**
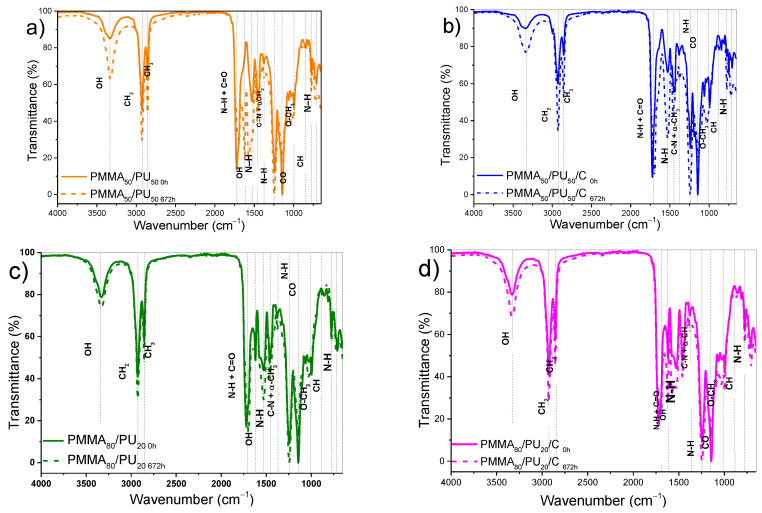
FTIR spectra of (**a**) PMMA_50_/PU_50_, (**b**) PMMA_50_/PU_50_/C, (**c**) PMMA_80_/PU_20_, and (**d**) PMMA_80_/PU_20_/C before (0 h) and after (672 h) aging exposure.

Unaged (PMMA_50_/PU_50_/C_0h_) and aged samples (PMMA_50_/PU_50_/C_672h_) were also analyzed by FTIR to confirm the presence of functional groups and to verify the absence of interference in the polymerization of PMMA and PU. The FTIR spectrum of the unaged sample, the pure PMMA_50_/PU_50_/C_0h_ sample, is shown in Figure 7a. The spectrum shows the characteristic band of the -OH vibrations at 3335 cm^−1^. Additionally, the typical methyl methylene groups of the PMMA are confirmed by the doublet between 2926 and 2986 cm^−1^, corresponding to the C-H stretching vibrations, and the bands at 1721 and 1143 cm^−1^ are associated with the C=O and -OCH_3_ stretching vibrations, respectively [40]. The vibration modes of PU are also evident. The bands at 1527 and 1228 cm^−1^ correspond to N-H bending vibrations of amide (II) and amide (III), respectively [15,41]. The band at 1453 cm^−1^ is attributed to the C-H scissoring and bending vibrations, while the bands at 847 and 781 cm^−1^ are related to the CH_2_ rocking vibration [42]. The PMMA_50_/PU_50_ spectrum after 672 h of irradiation exhibits significant changes due to aging. The characteristic band of -OH vibrations at 3335 cm^−1^ becomes more intense after aging due to the presence of humidity. In this regard, Kaczmarek et al. [36] note that the carbonyl region is important for observing changes produced by irradiation, particularly the bands at 1721 and 1143 cm^−1^, which are primarily used for this analysis. The intensity of the C=O and C-O bands at 1721 and 1145 cm^−1^ weakens following irradiation exposure, indicating the elimination of side groups [43]. Furthermore, the appearance of a new band at 1633 cm^−1^ can be attributed to the C=C stretching vibration, resulting from the rupture of C-H bonds in the composite [43]. FTIR analysis was also performed on the samples after adding 0.1 wt.% crystalline cellulose, as shown in Figure 7b (PMMA_50_/PU_50_/C_0h_). The difference between the pure sample and the composite sample is the reduction in the OH signal, which can indicate physical interactions between crystalline cellulose and PMMA and PU through hydrogen bonding, as well as a reduction in the intensity of the NH groups present in PU. These results confirm the successful polymerization of the two phases, PMMA and PU, in the presence of cellulose. Irradiation of PMMA_50_/PU_50_/C_672h_ provokes minimal changes, for example, a shift in the NH+CO signal from 1721 cm^−1^ to 1696 cm^−1^, likely due to the shortening of the polymer into shorter chains, while maintaining the other signals without modification. Additionally, the absence of the band at 1633 cm^−1^, associated with the C=C stretching vibration, is also noted. This result indicates that the addition of crystalline cellulose allows for avoiding C-H bond rupture in the sample after aging exposure. The functional groups of the PMMA_80_/PU_20 0h_ samples exhibit the same signals as those of PMMA_50_/PU_50 0h_, without modification, when crystalline cellulose is added (PMMA_50_/PU_50_/C_0h_). Similarly, OH vibrations are more intense after aging. Changes in the carbonyl region caused by radiation were also observed, with a shift from 1721 cm^−1^ to 1696 cm^−1^, regardless of whether crystalline cellulose was present or not. The presence of a C=C signal at 1619 cm^−1^ is more intense in samples with crystalline cellulose particles upon exposure to irradiation.

#### 3.2.2. Evaluation of Optical Transparency Using UV–vis Spectroscopy

**Figure 8:**
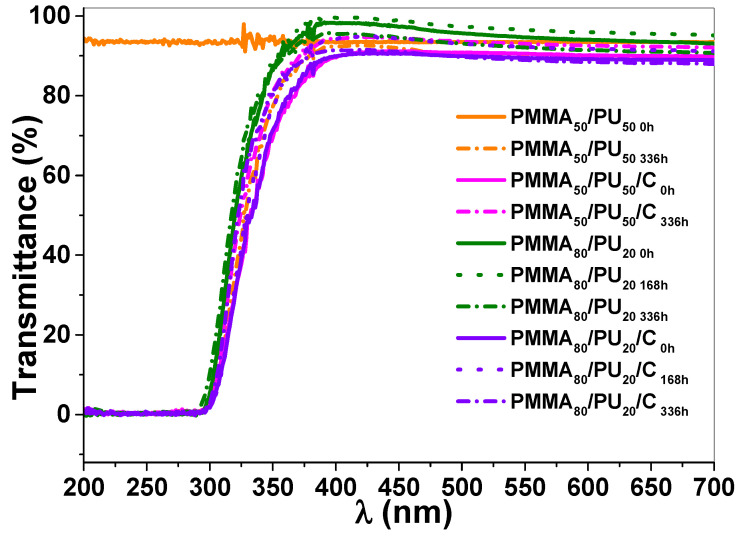
UV–vis spectra of selected-PMMA_50_/PU_50_, PMMA_50_/PU_50_/C, PMMA_80_/PU_20_, and PMMA_80_/PU_20_/C under aging exposure.

The optical transparency of PMMA_50_/PU_50_ films was examined using UV–Vis spectroscopy for both unirradiated and irradiated samples. The results are presented in Figure 8. Incorporating PU and crystalline cellulose does not affect the clarity of PMMA, as all samples are visually transparent in the visible range. The transmittance values for the PMMA_50_/PU_50 0h_ and PMMA_50_/PU_50_/C_0h_ samples at 320 nm (UV region) and 650 nm (visible region) are provided in Table 1.

The unirradiated PMMA_50_/PU_50 0h_ sample shows similar transmittance values in the UV region (99.8%) and the visible region (100%). PMMA exhibits high resistance to UV light exposure [44]. The PMMA_50_/PU_50 168h_ and PMMA_50_/PU_50 336h_ samples show no significant difference in their percentage of visible transmission at 650 nm (98.6 and 95.1%, respectively) after 168 h and 336 h of exposure. However, the aging process affects the transmittance values observed in the UV region at 320 nm, with values of 40.8 and 37.4, respectively. These results indicate that the aging process affects the structure of both phases due to the absorption in the UV region.

A decrease in the transmittance values at 320 nm is noted in the PMMA_50_/PU_50_/C_0h_ and PMMA_50_/PU_50_/C_336h_ samples after the addition of crystalline cellulose with values of 32.2 and 30.5%, respectively. The addition of cellulose has an effect in reducing the optical transparency, which is not considered so significantly since other systems have shown reductions up to 15% when cellulose nanoparticles are added into PMMA [45]. It is essential to consider that a PU single phase based on an HDI monomer has been reported to display transmittance values ca. 80–70% [46]. The PMMA_50_/PU_50_/C_0h_ and PMMA_50_/PU_50_/C_336h_ samples show 90.0 and 92.30%, respectively. Therefore, crystalline cellulose into PMMA/PU IPNs is modified slightly in the visible light protection of PMMA/PU/cellulose IPNs.

The optical transparency of the PMMA_80_/PU_20_ samples was also investigated using UV–vis spectroscopy, and the transmittance is presented in Table 1. A comparison between unirradiated samples (PMMA_50_/PU_50 0h_ and PMMA_80_/PU_20 0h_ samples) reveals that it presents a lower transmittance value at 320 nm (53.9%) than the second, which is due to the high content of PU. This sample exhibits high transmittance values between 94.4% and 90% in the visible region. The aging process did not affect the transmittance values in the UV region. The PMMA_80_/PU_20 168h_ and PMMA_80_/PU_20 336h_ samples exhibit intermediate transmittance values (50.9 and 56.2%) at 320 nm, which are close to the pure samples; the mismatch could be due to the thickness of the analyzed sample, although the same amount of polymer was used to evaluate it in this work. Future studies can be performed to evaluate the precise thickness on the transmittance value. On the other hand, the PMMA_80_/PU_20_/C_0h_, PMMA_80_/PU_20_/C_168h_, and PMMA_80_/PU_20_/C_336h_ samples present transmittance values of 30.3, 32.0, and 34.8%, respectively. These values are very similar to the PMMA_50_/PU_50_/C_0h_ samples. Therefore, the addition of crystalline cellulose modifies the UV region of the pure IPNs with a minimal effect on the visible region, which is essential for the appearance of the headlights. Then, it is suggested that chemical modifications be evaluated with molecules having UV-absorbing properties in crystalline cellulose that can enhance the optical properties of these samples.

#### 3.2.3. Tensile Behavior of PMMA/PU/Cellulose IPNs

**Figure 9:**
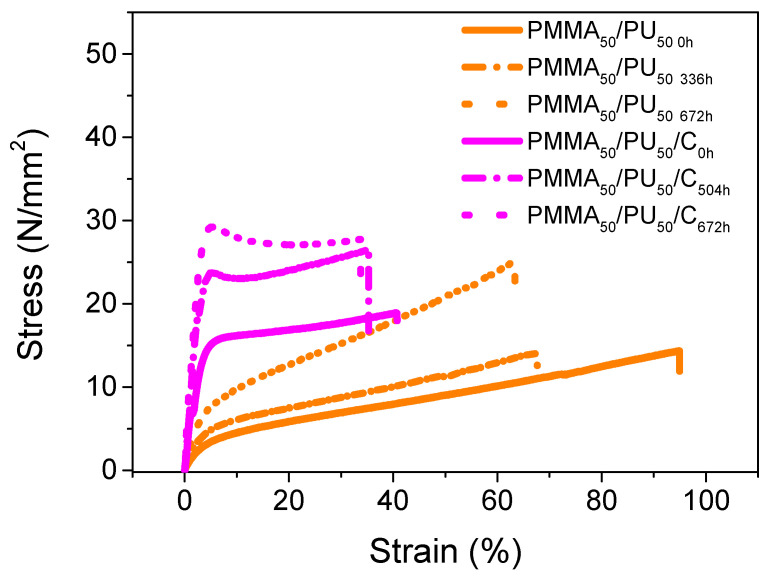
Tensile curves of PMMA_50_/PU_50_ and PMMA_50_/PU_50_/C before and after aging.

Figure 9 illustrates the tensile behavior of the pure sample, PMMA_50_/PU_50_, and the PMMA_80_/PU_20_/C_0h_, before and after undergoing the aging process. Table 2 summarizes the value. The PMMA_50_/PU_50_ curve exhibits a semi-ductile behavior of the polymer, achieving a strain of 95% with a tensile strength of 14 MPa and an elastic modulus of 83 MPa. After 336 h of aging, these values slightly decrease, with a tensile strength of 14 MPa—still similar to the pure sample—and a reduced strain percentage of 68%. Furthermore, the sample exhibits an impressive tensile strength of up to 25 MPa, with a minor reduction in the strain percentage to 63% and an increase in the Young’s modulus to 199 MPa. This behavior can be attributed to crosslinked chains due to the UV light from the sun. The addition of cellulose reinforces the tensile strength but diminishes the strain %. The PMMA_50_/PU_50_/C_0h_ specimens tend to be stronger than pure polymers. They display a maximum tensile strength of 19 MPa, with a strain % of 41% and an elastic modulus of 585 MPa. Compared to the pure sample, their ductility is reduced by 55%, resulting in a strain percentage of 40%, while the tensile strength increases to 18 MPa, consequently raising its Young’s modulus. The samples tend to have the behavior observed in the pure sample after 504 h of exposure; they become resistant with values of the tensile stress, Young’s modulus, and maximum strain % of 26 MPa, 751 MPa, and 35%, respectively. Finally, the tested samples retain their reinforcement after the aging process, demonstrating increased tensile strength and Young’s modulus values of 29 MPa and 892 MPa, respectively, with no significant modification in strain, which remains at 34%.

Reinforcing agents drastically modify the mechanical properties of polymers. As explained by S. Rahman et al. [47], the properties of the films are influenced not only by the weight percentage (wt%) of added particles but also by the type and nature of these particles. Different types of reinforcing agents, even if they share similar characteristics and matrices, can result in varying mechanical behaviors. This variation can also be due to the method used for their production and the percentage of fiber addition. In this study, compared to PMMA_50_/PU_50_, there is observed improvement in the tensile strength and Young’s modulus, although this comes with a decrease in elongation. Cellulose particles play a significant role in modifying the properties of the material in which they are dispersed. N. Jamaluddin et al. [48] have reported that the homogeneous dispersion of cellulose helps maintain the tensile strength values. Conversely, a deficiency of homogeneity leads to significant defects in the films, causing inconsistencies in the recorded data. In the same study, no improvement was found when cellulose underwent chemical modification, with the tensile strength and Young’s modulus showing only a 3% increase. Other research has indicated that adding ductile nanofibers, such as thermoplastic polyurethane (PU), into polymeric networks enhances the ductility of the material, which contrasts with the findings of this work. The discrepancy can be attributed to the chemical nature of cellulose and its various degrees of crystallinity, while PU is a ductile material [49]. Thus, by adding cellulose at a concentration of 0.1 wt%, an increase in tensile strength can be achieved, alongside an improved ability to maintain its deformation percentage when exposed to environmental elements.

**Figure 10:**
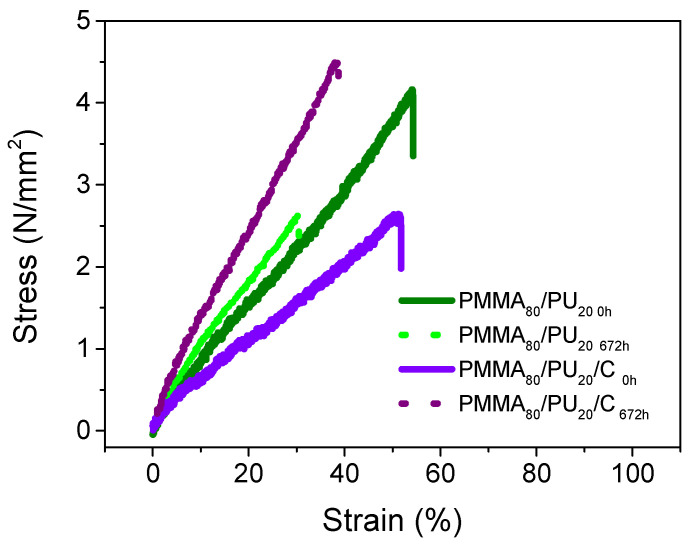
Tensile curves of PMMA_80_/PU_20_ and PMMA_80_/PU_80_/C before and after aging.

The effects of aging on samples with a high amount of PMMA (PMMA_80_PU_20_) are observed in Figure 10, along with the addition of cellulose (PMMA_80_/PU_20_/C); Table 3 provides a summary of the data.

The behavior of the pure sample shows a linear behavior associated with the elastic zone of the polymer. It exhibits a strain of 54%, a maximum tensile strength of 4 MPa, and a relatively low elastic modulus of 7 MPa. In comparison, this tensile strength is lower than the pure PMMA_50_/PU_50_ sample. Aging results in a reduction in the tensile strength to 3 MPa and a slight decrease in the strain percentage to 30%, while the Young’s modulus remains similar at 8 MPa. With the addition of cellulose particles, there is a minor decrease in the tensile properties, resulting in a strain of 51% and a tensile strength of 3 MPa, alongside an elastic modulus of 5 Mpa. The addition of these particles does not significantly detriment the properties. In fact, there is a reinforcement effect, as both the tensile strength and Young’s modulus increase with aging. J. Sethi et al. [50] discuss the behavior of cellulose when interacting with interpenetrating polymer networks of PU with poly(lactic acid) (PLA). Their findings suggest that concentrations equal to or less than 1 wt% of cellulose nanocrystals can enhance the mechanical properties, provided that adequate dispersion within the material is achieved. The study emphasizes the performance of PU, observing a reinforcement in elongation with values approaching 84%; however, it also notes a low Young’s modulus (7.4 Mpa), which is attributed to crosslinking among the hydroxyl groups of cellulose.

The data evaluated in this work demonstrate that the tensile properties are less affected in the pure samples compared to those with reinforced IPNs (PMMA/PU/C). In contrast, the damage is less severe in samples containing high amounts of PMMA (PMMA_80_/PU_20_/C) than those intermediate PMMA and PU (PMMA_50_/PU_50_/C).

#### 3.2.4. Compression Behavior of PMMA/PU/Cellulose Composites

**Figure 11:**
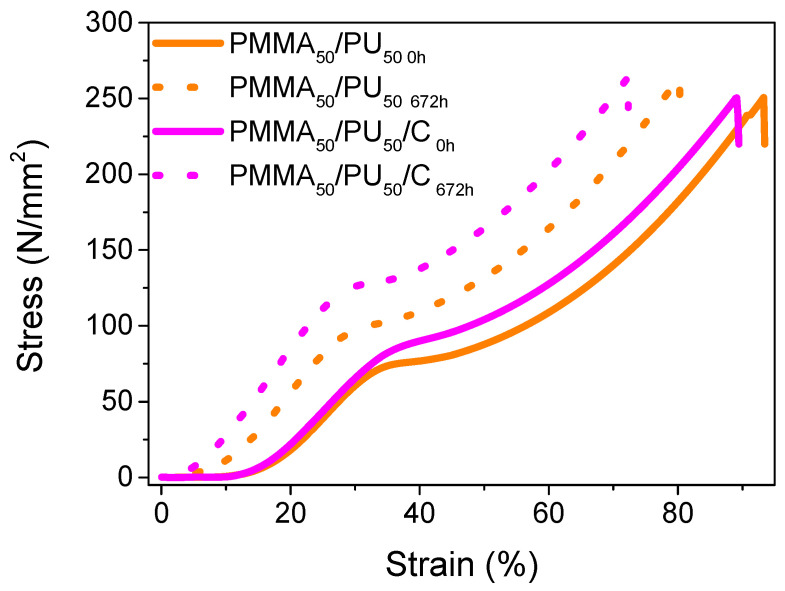
Compression curves of PMMA_50_/PU_50_ and PMMA_50_/PU_50_/C before and after aging.

The stress–strain curve for pure PMMA_50_/PU_50_ exhibits a typical three-phase curve between 8 and 20% strain (see Figure 11 and Table 4). The initial section of the curve indicates elongation without any permanent strain in the material, which is characteristic of the elastic region of the polymer. As the strain reaches 35%, the materials enter the plastic zone, leading to a permanent alteration in the polymer until a maximum strain of 91% is achieved. At this point, the material is fully compacted without showing signs of rupture, and the Young’s modulus is estimated at 7 MPa. The properties change slightly after 672 h. There is only a slight increase in the strain from 12% to 18%.

The addition of cellulose does not significantly change the properties of the pure sample, as it exhibits very similar values: a tensile strength of 249 MPa, a strain of 89%, and a slight increase in the Young’s modulus to 13 MPa. Samples containing cellulose exhibit a more stable behavior. Notably, these samples display a broader range of performance in the elastic phase of the material, indicating that they can support greater loads without suffering permanent strain. This behavior aligns with expectations for cellulose as a reinforcing agent. D. Mendoza et al. [51] manage to demonstrate a relationship between the reinforcing properties of cellulose and the enhancement in the mechanical properties of multiple materials created through IPNs. It is evident that the performance can vary based on differences in the crosslinking ratio of the networks and the inclusion of cellulose, achieving mechanical property values up to 10 times higher compared to pure components. Finally, the compression properties measured after 672 h show low strain (73%), along with a high tensile strength (264 MPa) and Young’s modulus (19 MPa).

**Figure 12:**
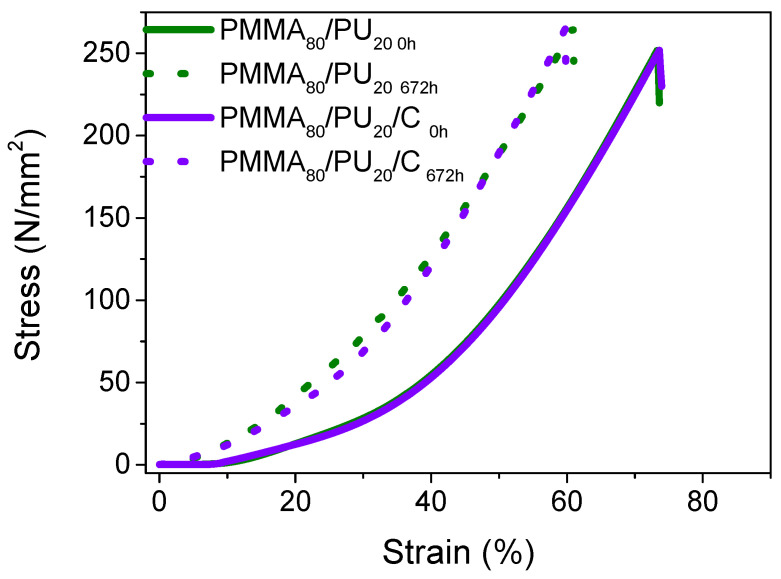
Compression curves of PMMA_80_/PU_20_ and PMMA_80_/PU_80_/C before and after aging.

In the cases of the PMMA_80_/PU_20_ and PMMA_80_/PU_20_/C films (see Figure 12 and Table 5), a noticeable behavior change occurs as the exposure time increases. The observed curves display asymptotic behavior right from the beginning. M. Piñero et al. [52] attribute these characteristics to the material and its properties, highlighting its nature as an elastomer with a high degree of crosslinking in the chains, allowing for a deformation capacity of up to 50%.

The PMMA_80_/PU_20_ samples exhibit a compressive strength of 250 MPa and a strain of 74%, with a notable Young’s modulus of 3.12 MPa. Compared to those reinforced with cellulose, it has a compressive strength of 249 MPa and a strain of 81%, in addition to a Young’s modulus of 3.47. This indicates that the addition of cellulose does not enhance reinforcement. Additionally, it was noted that the tensile strength properties of the samples tend to be higher than those of the pure samples, displaying a tensile strength of 265 MPa, a strain of 60%, and a Young’s modulus of 6–7 MPa. These measurements show no significant differences when compared to the cellulose-added samples after 672 h of exposure.

No commercially available PMMA/PU or PMMA/PU/C IPN-based plastics are found for headlight comparison in automotive applications. Some research has been conducted using individual PMMA and PU in thermoplastic form for instrument panels [53,54], as well as laminates based on PMMA with thermoplastic PU for car headlights [55]. In the latter case, the authors mention a transparent material; however, there is no evidence of studies to corroborate this claim [55]. Other research has simulated components of a headlamp, including polycarbonate (PC), polybutylene terephthalate (PBT), PPTD40 (Toyota, Toyota City, Japan), carbon fiber-reinforced polyester (BMC 1000, Lyondell Basell, Rotterdam, The Netherlands), and polyoxymethylene (POM). The reported Young’s moduli for these materials are approximately 2350, 2400, 3500, 13,000, and 3000 MPa, respectively [56], which are significantly different from the data presented in this study. It is essential to note that the reported data corresponds to the full headlight assemblies, while this study focuses on films with a thickness of 4 mm. The IPNs discussed here display transmittance values that are very similar to and even better than those of commercial headlights using halogen, LED, and xenon technologies, typically displaying a transmittance of around 20% [57].

Then, this study highlighted the importance of integrating crystalline cellulose into materials that will undergo stresses, as this can greatly improve their performance in dependence of the PMMA/PU ratio. It also investigated how these materials maintain their advantageous properties when subjected to aging conditions, ultimately extending their useful lifespan. In this sense, X-ray diffraction analytical techniques, FTIR, and CLSM were essential for identifying the properties of both crystalline cellulose and its dispersion in the PMMA/PU IPNs. Each has its specific strengths, and their combined use enables an integrated vision of the structure and composition of the materials, facilitating advances in research and development and industrial applications, such as coatings for automotive headlights.

Based on these findings, incorporating crystalline cellulose into PMMA/PU enables the polymer to retain its properties during exposure to UV light and environmental humidity. For these reasons, it presents potential properties for use as a coating in automotive headlights, avoiding structural deterioration and extending the useful life. However, expanding the study for more than 674 h under natural aging is advisable.

## 4. Conclusions

The proposed methodology to valorize and recycle single-use cups for cold beverages was efficacious in extracting crystalline cellulose. The pretreatments employed to remove extractives, and amorphous cellulose did not modify the cellulose structure, enabling the obtaining of nanosized biopolymer.

The extracted cellulose biopolymer consisted of a mixture of polymorphs, Iα, and Iβ, with intermediate crystallinity. It can be easily dispersed without affecting the polymerization of PMMA and PU to obtain interlocking networks.

The effect of the concentration on the tensile properties indicates that adding a more significant amount of PU (PMMA_50_/PU_50_) reinforces the strength, allowing a highly ductile polymer. Crystalline cellulose nanoparticles tend to reinforce stress and Young’s modulus when added to a similar ratio of phases (50/50, PMMA_50_/PU_50_) in the tensile properties. In contrast, the values are reduced in the presence of a high amount of PMMA (80/20, PMMA_80_/PU_20_). The compression properties are not affected significantly when cellulose is added.

The natural aging process indicates less tensile damage when adding crystalline cellulose, especially in the PMMA_50_/PU_50_/C samples compared to the PMMA_80_/PU_20_ samples. The compression and optical properties are not modified significantly when aging.

This work promotes the reduction in the environmental impact of disposable cups through a sustainable alternative to obtaining cellulose nanoparticles and producing advanced polymer compounds, which have potential use in automotive headlights.

## Figures and Tables

**Table 1:** Transmittance percentages of PMMA_50_/PU_50_, PMMA_50_/PU_50_/C, PMMA_80_/PU_20_, and PMMA_80_/PU_20_/C at 320 and 650 nm during aging up to 336 h.

Sample	Transmittance (%)	
	320 nm	650 nm
PMMA_50_/PU_50 0h_	99.8	100
PMMA_50_/PU_50 168h_	40.8	98.6
PMMA_50_/PU_50 336h_	37.4	95.1
PMMA_50_/PU_50_/C_0h_	32.2	90.0
PMMA_50_/PU_50_/C_336h_	30.1	92.3
PMMA_80_/PU_20 0h_	53.9	100
PMMA_80_/PU_20 168h_	50.9	100
PMMA_80_/PU_20 336h_	56.2	97.4
PMMA_80_/PU_20_/C_0h_	30.3	95.2
PMMA_80_/PU_20_/C_168h_	32.0	91.4
PMMA_80_/PU_20_/C_336h_	34.8	88.2

**Table 2:** Tensile data for PMMA_50_/PU_50_ and PMMA_50_/PU_50_/C before and after aging.

Sample	Stress (MPa)	Strain (%)	Young’s Modulus (MPa)
PMMA_50_/PU_50 0h_	14.18 ± 0.29	94.92 ± 0.03	82.83 ± 0.08
PMMA_50_/PU_50 336h_	13.54 ± 0.12	67.62 ± 0.12	152.50 ± 0.36
PMMA_50_/PU_50 672h_	25.01 ± 0.17	63.14 ± 0.23	198.50 ± 0.78
PMMA_50_/PU_50_/C_0h_	18.78 ± 0.12	40.73 ± 0.03	585.19 ± 0.21
PMMA_50_/PU_50_/C_504h_	26.48 ± 0.46	35.10 ± 0.04	750.71 ± 0.29
PMMA_50_/PU_50_/C_672h_	29.24 ± 1.12	33.59 ± 0.23	891.71 ± 0.56

**Table 3:** Tensile data for PMMA_80_/PU_20_ and PMMA_80_/PU_20_/C before and after aging.

Sample	Stress (MPa)	Strain (%)	Young’s Modulus (MPa)
PMMA_80_/PU_20 0h_	3.76 ± 0.37	54.20 ± 0.08	7.23± 0.36
PMMA_80_/PU_20 674h_	2.60 ± 0.72	30.44 ± 0.29	7.99 ± 0.85
PMMA_80_/PU_20)_/C_0h_	2.59 ± 0.04	51.17 ± 0.38	4.74 ± 0.45
PMMA_80_/PU_20_/C_674h_	3.14 ± 0.73	40.94 ± 0.23	10.92 ± 0.94

**Table 4:** Compression data for PMMA_50_/PU_50_ and PMMA_50_/PU_50_/C before and after aging.

Muestra	Stress (MPa)	Strain (%)	Young’s Modulus (MPa)
PMMA_50_/PU_50 0h_	249.70 ± 0.97	93.10 ± 0.29	7.45 ± 0.03
PMMA_50_/PU_50 672h_	264.59 ± 0.31	80.35 ± 0.12	9.91 ± 0.09
PMMA_50_/PU_50_/C_0h_	249.87 ± 0.66	89.27 ± 0.16	13.21± 0.46
PMMA_50_/PU_50_/C_672h_	264.08 ± 0.27	72.65 ± 0.56	18.55 ± 0.45

**Table 5:** Compression data for PMMA_80_/PU_20_ and PMMA_80_/PU_20_/C before and after aging.

Sample	Stress (MPa)	Strain (%)	Young’s Modulus (MPa)
PMMA_80_/PU_20 0h_	249.97 ± 1.31	73.51 ± 0.21	3.12 ± 2.84
PMMA_80_/PU_20 674h_	265.37 ± 0.37	60.94 ± 1.29	6.33 ± 0.06
PMMA_80_/PU_20)_/C_0h_	249.41 ± 1.23	74.00 ± 0.12	3.47 ± 1.27
PMMA_80_/PU_20_/C_674h_	265.05 ± 0.77	59.43 ± 0.50	7.60 ± 0.42

## Data Availability

The original contributions presented in this study are included in the article. Further inquiries can be directed to the corresponding author.
